# Development of an energy-efficient indirect type dryer using a triangular duct solar air heater with the synergistic effect of pin-fins and discrete V-ribs: A 3-D CFD study

**DOI:** 10.1038/s41598-025-31053-7

**Published:** 2025-12-03

**Authors:** Mohan Sushmitha, Pranshu Gadepalli, Kottayat Nidhul

**Affiliations:** Renewable Energy Center, Department of Mechanical and Industrial Engineering, Manipal Institute of Technology, Manipal Academy of Higher Education, Manipal, 576104 Karnataka India

**Keywords:** Energy-efficient dryer, Triangular duct solar air heater, Thermo-hydraulic performance parameter, Pin-fins, V-ribs, Indirect type solar dryer, Energy science and technology, Engineering, Physics

## Abstract

A high-performance triangular duct solar air heater (TSAH) incorporating discrete V-ribs and pin-fins is numerically analysed to enhance thermo-hydraulic and drying performance for indirect-type solar dryers (ITSD). Three-dimensional CFD simulations are performed for 5000 ≤ Re ≤ 15,000, exploring the effects of pitch ratio (P/e = 5–15), gap width (g/e = 0.2–0.8), and solid/porous pin-fins. Introducing a gap at the V-rib apex increases local turbulence and secondary flow interactions, yielding a Nusselt number enhancement of 3.36 and a friction factor increase of 2.98 for g/e = 0.4 at Re = 5000, resulting in a THPP of 2.35. Integrating porous pin-fins further enhances vortex mixing and thermal interaction, achieving a maximum THPP of 2.68, which surpasses the existing rib geometries. When applied to mushroom drying in an ITSD, the enhanced TSAH delivers higher outlet air temperatures and stronger secondary flow intensity, leading to a 66% reduction in drying time and a 54% increase in effective diffusivity compared to a smooth-plate SAH. The results demonstrate that combining discrete V-ribs with porous pin-fins offers a synergistic improvement in heat transfer, pressure drop balance, and drying kinetics, enabling energy-efficient drying applications.

## Introduction

Post-harvest losses of fruits and vegetables, especially in developing countries, significantly threaten global food security, with up to 40% of produce lost between harvest and consumption. Traditional open sun drying (OSD), though cost-effective, often leads to poor product quality due to contamination and uncontrolled conditions. Solar drying offers a sustainable alternative by utilising free solar energy, providing better control over the drying process and reducing contamination risks. Indirect solar dryers (ITSDS) are particularly suitable for sensitive crops, as they prevent direct exposure to sunlight and allow for improved temperature control. Enhancing the heat transfer efficiency of solar air heaters is crucial for optimising the performance of these solar drying systems. Solar air heaters (SAHs) are integral elements within renewable energy systems. They are crucial for harnessing solar energy for multiple applications, such as in agriculture for drying purposes^1,2^. Over the years, different approaches, such as numerical and experimental methodologies, were used to optimise the design of SAH^3^, leading to increased thermal performance, energy efficiency, and exergy utilisation. Several factors influence the effectiveness of solar air collectors, such as the amount of solar radiation incident on them, the angle of the flat plate, the design of the collectors, and the airflow pattern^4^. Research findings indicated that a peak flow rate was attained at an inclination angle of 60°, resulting in an incident radiation increase of approximately 17%^5^. It has been determined that the time taken for air to circulate within the heater is critical^6^.

Duct configuration is important for optimising the effectiveness of SAH. While rectangular ducts are commonly used for traditional applications, triangular ducts offer distinct advantages. When a comparative performance analysis was conducted between a triangular duct cross-section and a rectangular duct cross-section, SAH with similar rib configurations ensuring consistent heat input and flow Re, an overall performance enhancement was seen in triangular duct configurations^7^. The equilateral triangular shape reduces the duct volume and ensures a uniform distribution of heat transfer. Due to the formation of a laminar sub-layer underneath or above the absorber plate, the convective heat transfer rate decreases along its length, depending upon the duct design. Incorporating ribs changes the flow pattern, which alters the fluid’s thermal and hydraulic behaviour within SAH^8,9^. The effectiveness of dimple-shaped rib roughness is limited at low Reynolds numbers (Re)^10^. The maximum friction factor enhancement ratio (FFER) of 2.97 was at a Re of 4 × 10^3^ in pentagonal and trapezoidal ribs when compared with the base model of a flat plate SAH^11,12^. Furthermore, the efficiency of the absorber plate with a conical pin was enhanced by up to 26.5%^13^ and by 3–12% with a pin fin compared to a flat smooth plate^14^. Although both pins and ribs improve performance, pin fins are superior for localised heat transfer, while ribs offer a more extensive and uniform enhancement. The transverse rib considerably enhances the heat transfer rate due to the formation of vortices, which increase the surface area for heat transfer^15^. In a comparative analysis, the parallel pass hybrid duct SAH with inclined ribs, compared with a traditional rectangular duct parallel pass SAH, showed enhancements in thermal efficiency of 18.1%^16^. Notably, ribs with 45° inclination in the triangular duct achieve a 17% higher effectiveness than the conventional model^17^. Moreover, an absorber plate with fins demonstrates a thermal efficiency improvement ranging from 7.3% to 25.8% over conventional air heaters^18^.

The V-shaping of long angled ribs generates two secondary flow cells, unlike the single cell formed by a standard angled rib, which results in an increased heat transfer rate^15^. SAH with a triangular duct configuration consisting of V-Ribs to facilitate temperature increases, impacting even the innermost section of the duct^19^. Implementing a gap within the inclined rib has been noted to improve heat transfer efficiency by disrupting the secondary flow and increasing turbulence created downstream of the rib in the fluid^15^. A numerical investigation of a triangular duct SAH featuring V ribs revealed that the peak heat transfer rate occurs at an angle of α = 45⁰, independent of the Reynolds number^20^. The Nusselt number and friction factor are higher for the multi-V-shaped rib with a gap than for the continuous variant^21^. It is also observed that the thermo-hydraulic performance parameter (THPP) enhances as the angle of attack of the flow increases^22^. According to the above studies, the convective heat transfer coefficient between the absorber plate and the air can be significantly enhanced by incorporating artificial roughness on the collector plate^23^.

With dimple-shaped cavities underneath the absorber plate, it is observed that higher heat transfer occurs at the leading edge than on the trailing edge^24^. In another numerical study, circular and triangular geometries enhanced the heat transfer rate by 2.18 and 2.35 times, respectively, compared to a smooth duct^25^. Furthermore, introducing delta-shaped obstacles within the channel resulted in backflow between the obstructions and the elevated velocity in the gaps. This led to increased turbulence and improved convective heat transfer between the air and the collector plate^26^. Additionally, when arcs were utilised as roughness in an exergy analysis, thermal efficiency rose with increasing Reynolds numbers, attributed to improved convective heat transfer rate^27^. With transverse ribs, energy losses and glass side Nusselt number are lower resulting in 24% improvement over smooth plate SAH^28^. The inclined corrugated grooves on the absorber surface significantly influence the flow, generating longitudinal swirl flow within the air heater duct, facilitating better air mixing, and enhancing heat transfer^29^. With perforated baffles of varying heights, a THPP of 2.54 was observed in a V-down configuration^30^.

The performance of finned and corrugated absorber plates was compared using experimental and theoretical approaches to enhance the efficiency of traditional air heating systems with flat plates. The outcomes suggested that the v-corrugated collector attains an efficiency enhancement of 10% to 15% during its single-pass operation^31^. A comparative analysis of the tubular SAH against the traditional flat plate SAH (FSAH) demonstrated that the tubular SAH consistently outperforms the FSAH in terms of outlet air temperature, heat gain, and overall efficiency across all tested inlet airflow rates, while also exhibiting reduced top heat losses. However, it is noted that the pressure drops associated with the tubular SAH are higher than those of the flat SAH^32^. An experimental evaluation of two types of corrugated absorbers alongside a flat plate collector revealed that both corrugated designs significantly outperformed the flat plate, achieving efficiencies of 58.9% for type 1, 60.3% for type 2, and 48.6% for FSAH^33^. A V-groove solar air heater with transverse wedge-shaped ribs achieved maximum thermal performance at P/e = 8 and e/D_h_ = 0.07^34^. With V-ribs of 60° and P/e = 8, a 16% higher thermal efficiency compared to a smooth plate solar air heater was obtained for e/Dh = 0.043^35^. A maximum THPP of 1.97 was obtained with hollow semi-stadium fins, amounting to 97% enhancement compared to smooth plate SAH^36^. With similar fin configuration, double pass solar air heater achieved a maximum thermal efficiency of 78% compared to smooth plate SAH with an enhancement of 31%^37^.

An analysis study reveals that porous flat plate collectors can achieve substantial performance improvements compared to similar non-porous models for similar optical properties and top heat loss^38^. Additionally, the potential for performance enhancement can be as high as 102% in the analysis of non-porous types^39^. Another study with porous SAH reported thermal efficiency improvement from 10% to 20%^40^. Non-uniform porous medium in the form of ascending and descending configurations resulted in better thermal and collector efficiency compared to a uniform porous medium^41^.

An indirect-type solar dryer (ITSD) consists of a SAH and a drying cabinet wherein the specimens to be dried are kept. As ITSD can contribute to reducing post-harvesting losses, especially in developing countries, the design of SAH, an integral part, plays a crucial role in achieving better performance with minimal operating costs. While reporting that ITSD has demonstrated superior performance in terms of speed and quality of drying, computer simulations would aid in analysing the performance before implementing it on a large scale^17,42^. For industrial scale drying, it was reported that ITSD with forced convection mode has higher drying rates than natural convection mode^43^. While mentioning the effect of essential drying factors, such as inlet air velocity, temperature, and humidity of air^43^, it was also reported that a cost-effective approach is required for implementing dryers, considering both capital and operational costs for integrating solar-based technologies^44^. It was also suggested that enhancing the absorber surface geometry in SAH would improve the convective heat transfer rate^45,46^, thereby achieving higher drying efficiency.

As SAH is an integral part of ITSD, its design is vital in drying performance and, in turn, to minimise the drying cost. In this regard, TSAH with discrete V-ribs has been studied along with pin-fins in the inter-rib region to analyse the synergistic effect of secondary flow and pin-fins on thermo-hydraulic performance. Even though hybrid rib configurations have been studied in flat plate SAH, the same has not been explored in depth in triangular duct SAH, especially from the secondary flow perspective. Initially, the pitch varies for a fixed rib height and inclination to reach the optimal pitch. With the optimal pitch, the effect of gap width at the apex of the ribs is studied, and with the optimal gap width, the effect of the presence of pin-fins (having a diameter and rib height equal to rib height), solid and porous, in the region of secondary flow coalescence is studied. Furthermore, as the proposed design exhibits higher thermo-hydraulic performance parameters than the existing TSAH designs, it is proposed for ITSD. The studies on ITSD with triangular duct are meagre and need to be studied owing to the higher surface-to-area ratio of the triangular duct SAH. The drying performance of the ITSD with the new SAH design is studied in terms of moisture ratio, drying rate, and equilibrium diffusion coefficient variation over time, and compared with the ITSD having a smooth plate SAH for mushroom food samples.

## Numerical analysis

### Description of the problem

**Fig. 1:**
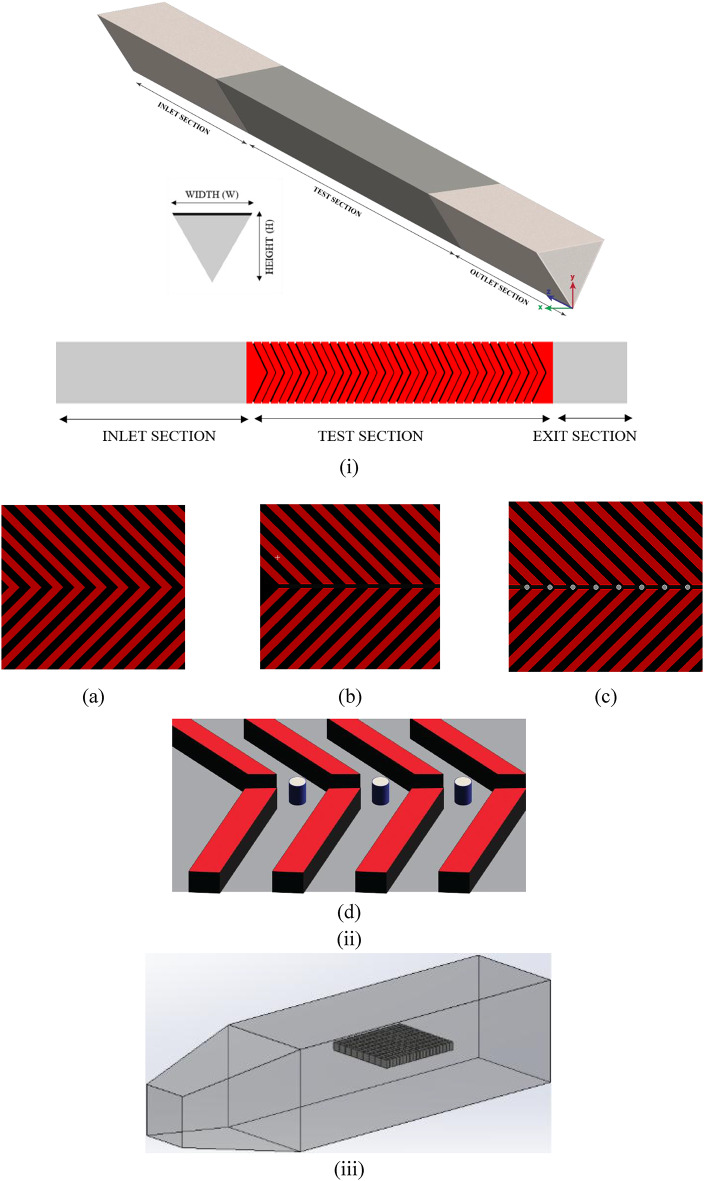
(i) Schematic diagram of the triangular duct, (ii) (**a**) V rib configuration, (**b**) V rib configuration with gap, and (**c**) V rib configuration with gap and pin fins, (**d**) Isometric representation of discrete V-rib with pin fin (iii) Drying cabinet with food samples at the centre (flow direction is from left to right).

V ribs of the square cross-section are employed in a solar air heater (SAH) with a triangular duct, as shown in Fig. 1(i)(ii). The duct has a height of 65.8 mm and a cross-section of an equilateral triangle with a side length of 76 mm and an apex angle of 60 °. For a fixed rib height (e) of 5 mm and rib inclination (45°), relative roughness pitch (P/e) is varied in the range of 5 to 15. Furthermore, the impact of gap width (g) is investigated by varying g/e (1 to 4 mm), and pin-fins in the inter-rib region are examined. A test section of 800 mm is chosen with an entrance of 5√(WXH) and an exit section of 2.5√(WXH) to obtain a thermally established flow with minimal impacts. The V-rib triangular duct SAH is modelled using Ansys Design Modeller v19.0. The computational domain consists of three sections: an entrance, a test, and an exit section, all adhering to the specifications outlined in ASHRAE standard 93 − 77^45^. The V ribs are modelled beneath the absorber plate. For the turbulent flow regime, the Reynolds number ranges from 5000 to 15,000 for a hydraulic diameter of 0.044 m. The Re is chosen such that the flow is turbulent in nature, allowing for the extraction of heat from the hot absorber plate while also avoiding excessive pressure drop, which could arise with higher Re, thereby offsetting the gains from enhanced heat transfer. Furthermore, as a solar air heater is an integral part of an indirect-type solar dryer, a higher flow rate would reduce the residence time of air within the air heating unit, causing a lower outlet air temperature. A consistent heat flux of 1000 W/m² is directly applied to the collector plate^8^. The dryer geometry consists of a chamber with a convergent outlet to prevent backflow. Food sample, mushroom slices are in the form of cuboids as shown in Fig. 1 (iii). The mushrooms are placed midway through the length of the duct at the centre on a thin tray. The dimensions of the dryer are mentioned in Table 1.

**Table 1:** Dryer dimensions.

Domain	Dimensions [L x W x H]	Units
Dryer (rectangular part)	1000 × 320 × 320	mm
Dryer (convergent part)	400 × 160 × 160	mm
Dimensions of a food sample (180 samples)	25.8 × 12.9 × 25.8	mm

### Grid generation

**Fig. 2:**
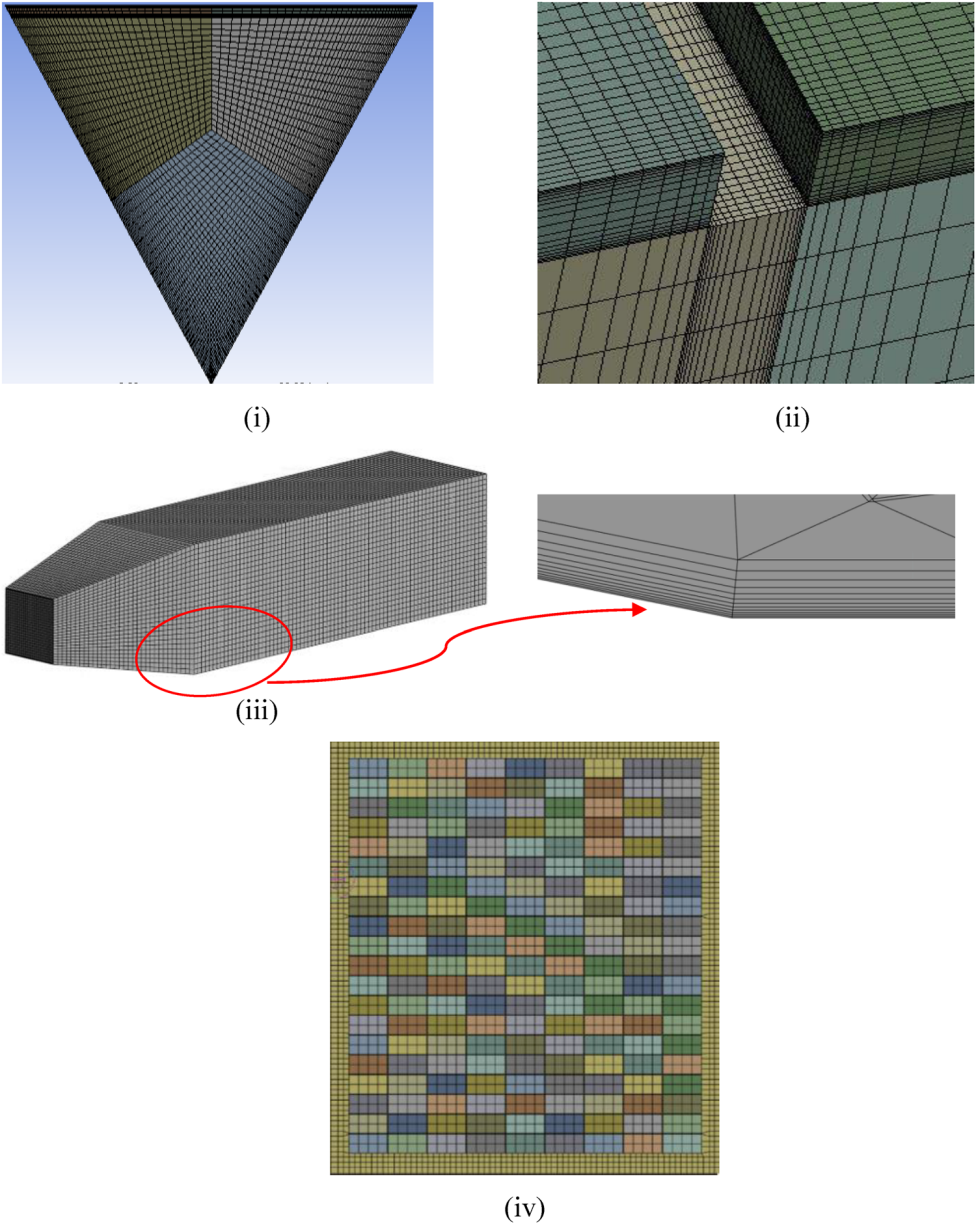
(i) Discretisation of duct cross-section using hexahedral cells using a multiblock meshing approach, (ii) Magnified image of mesh close to the walls of the rib, (iii) Discretisation of drying cabinet (with inflation layers), and (iv) Discretisation of food sample.

**Fig. 3:**
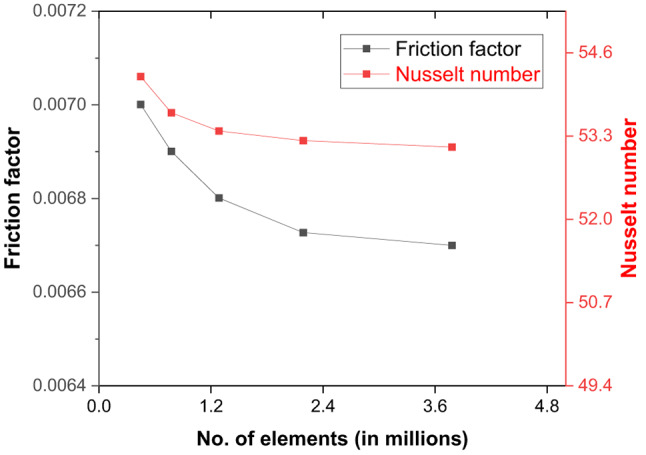
Grid independence study for triangular duct solar air heater.

A multi-block meshing approach generates the non-uniform structured grid using edge sizing and an appropriate biasing option for a high-density grid with y^+^ ≈ 1 that can capture the intricate flow details within the laminar sublayer near the ribs and gaps within the ribs (Fig. 2). A grid independence test is executed to evaluate the reliability of the results for variations in grid size. Measurements of the Nusselt number (Nu) and friction factor (f) are obtained at a Reynolds number (Re) of 15,000 for various sizes of grids to identify the most effective grid size. The findings indicated that grid sizes larger than 2.4 × 10^5^ elements exhibited a less than 0.5% disparity for Nu and f, thus justifying the selection (Fig. 3). In addition, the grid independence is verified using the proposed grid convergence index (GCI) method^46^. In addition, the grid independence is verified using Richardson’s extrapolation so that the results do not vary with the spatial discretisation of the domain. With the mesh comprising 1,284,192 elements, the error estimated for *Nu* and *f* with the GCI method is 0.7% ad 1.2%, espectively.

The dryer computational domain is discretised using an unstructured grid comprising hexahedral and tetrahedral elements. 12 prism layers were added with a first layer thickness of 0.01 mm to accurately capture the boundary layer velocity profile, as shown in Fig. 2(iii), which resulted in a wall y + of 0.4. The mesh in the region near the mushrooms is refined to ensure that the gaps between individual mushrooms are captured without compromising mesh quality. A grid independence study was conducted, and the satisfactory mesh controls have been listed in Table 2. The mesh metrics shown in Table 3 validate the mesh as the required cell quality and skewness are within the permissible limits for a CFD computation.

**Table 2:** Mesh parameters of the dryer unit.

Domain	Method	Size
Dryer (rectangular part)	Multizone	13 mm
Dryer (convergent part)	Multizone	13 mm
Refinement region	Cartesian	4 mm
Mushroom slices	Cartesian	7 mm

**Table 3:** Quality metrics of the grid generated for the dryer.

Mesh metric	Minimum	Maximum	Average
Orthogonal quality	0.17	1	0.76
Skewness	0	0.83	0.29

### CFD analysis

Conducting a three-dimensional computational fluid dynamics (CFD) simulation is essential as the secondary flow can be effectively represented along the rib length. The thermo-physical properties of air are considered to vary with temperature and implemented in the simulation as a user-defined function (udf). Several assumptions have been used for the present numerical study, which are as follows. The flow remains incompressible throughout the turbulent regime with steady-state conditions. Fluid properties are uniform throughout the duct length. As the walls of the duct exhibit a level of smoothness, a “no-slip” and “no-temperature jump.” Further, the inlet, test, and outlet sections are isothermally insulated. The test section length chosen is sufficient for the flow to be thermally developed, with the inlet and exit sections ensuring hydrodynamically developed flow and negligible end effects, respectively^8,47^.

The boundary conditions established for the three-dimensional domain are specified as follows:

I. A velocity between 1.83 and 7.31 m/s is applied at the inlet of the domain, and the temperature of the ambient air is taken as 30 °C.
II. Outlet is maintained at a pressure of 101.325 kPa.
III. A heat flux of 1000 W/m² is incident on the absorber plate made of aluminium.
IV. All remaining domain walls, apart from the absorber plate, are considered adiabatic and subject to a no-slip condition.

The determination of average flow velocity is achieved by using the Reynolds number. The governing equations (continuity, momentum, and energy) are subjected to discretisation via the SIMPLE algorithm alongside a second-order upwind scheme.

**Fig. 4:**
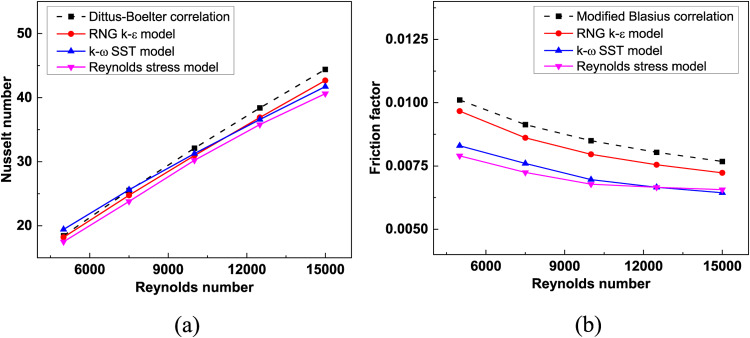
Estimation of (**a**) Nusselt number and (**b**) Friction factor by various turbulence models for smooth triangular duct SAH.

In turbulent flow modelling, the proper selection of a turbulence model is of prime importance for the results to be reasonably accurate. The flow problem is simulated using various turbulence models to estimate Nu and f for varying Re, as shown in Fig. 4 (a) and (b), respectively. With an average deviation of 2.5% and 8.4%, respectively, the RNG k-ε turbulence model with enhanced wall treatment (EWT) provides reasonably accurate results compared to the rest^48^. With continuity and energy residuals set to 10^− 6^ and 10^− 10^, respectively, the average temperature of the collector plate and the pressure drop that occurs in each iteration are monitored for the attainment of a steady state.

The local thermal equilibrium (LTE) can be considered for this problem, as the temperature difference between the fluid and solid structure is low^49^, and the energy equation is given by

1 $$\:{\left(\rho\:{c}_{p}\right)}_{f}\left(\overrightarrow{\text{u}}\phi\:\nabla\:T\right)=\left({k}_{e}\right){\nabla\:}^{2}T$$

Where, $\:{k}_{e}\:$is the effective thermal conductivity of solid and fluid regions and is given by:

2 $$\:\:\:\:\:\:\:\:\:\:\:\:\:\:\:\:\:\:\:\:\:\:\:\:\:\:\:\:\:\:\:\:\:\:\:\:\:\:\:\:\:\:\:\:\:\:\:\:\:\:\:\:\:\:\:\:{k}_{e}=\left(1-\phi\:\right){k}_{s}+\:\epsilon\:{k}_{f}$$

The porous medium used in this study is Aluminium Oxide $\:{(Al}_{2}{O}_{3})$ has significant applications in high-temperature regions^49,50^.

The parameters used in this study are detailed in Table 4.

**Table 4:** Properties of the porous media.

Porous Parameters	Value
Thermal Conductivity ($\:{k}_{e}$) (W/m. K)	9.78
Porosity $\:\left(\phi\:\right)$	0.918
Pore Density	10 PPI
Inertia Coefficient ($\:{C}_{F}$)	$\:0.027$
Permeability (K) ($\:{m}^{2})$	$\:1.98\:\times\:{10}^{-8}$
Pore diameter ($\:{d}_{p})$ (m)	$\:2.03\:\times\:{10}^{-3}$

The Ergun equation^51^ is used to calculate the viscous resistance and inertial resistance for fluid in the porous medium and is given by:

Viscous Resistance:

3 $$\:K=\frac{{d}_{p}^{2}}{150}\frac{{\phi\:}^{2}}{{(1-\phi\:)}^{2}}$$

Inertial Resistance:

4 $$\:{C}_{f}=\frac{3.5}{{d}_{p}}\frac{(1-\phi\:)}{{\phi\:}^{3}}$$

Further 3D CFD studies are carried out to explore the variation in drying rate, moisture content, and effective diffusion coefficient of the food sample within the ITSD. Sufficient iterations were provided for the solution to converge at every time step. The CFL (Courant–Friedrichs–Lewy) number is maintained below 5 throughout the simulation to ensure stability of the solution. Other parameters, such as drying rate and moisture content, are also monitored throughout the transient simulations.

A total of 180 mushroom food samples of given dimensions are considered as a porous medium. The inlet velocity and temperature were equal to the outlet temperature and velocity of air at the outlet of the solar air heater, and an atmospheric pressure outlet boundary condition was employed. The relative humidity of the ambient air is 90%. The material properties of mushrooms are considered constant, as temperature variation is not significant (Table 5).

**Table 5:** Material properties and boundary condition^52^.

Property	Value	Unit
Mushroom density	769	kg/m^3^
Mushroom thermal conductivity	0.374	W/m.K
Mushroom specific heat	2.960	kJ/kg.K
Mushroom porosity	0.38	-
Initial Moisture Content	0.93	-

Since ANSYS Fluent does not have a model for analysing drying, the calculations are performed with user-defined functions (UDF). These equations are written in the C programming language using macros available in Fluent, and the code is compiled within Fluent. Three main macros were used to write the UDF:.

DEFINE_INIT(name, d) – Used to assign the initial value of the variables.

The DEFINE_ADJUST macro is executed by Fluent before every iteration or time step, and is therefore used to update intermediate physical quantities that depend on the current flow field. In our drying model, this macro was responsible for evaluating the local drying rate based on temperature and moisture content, updating the moisture content field stored in user-defined memory (UDM), and preparing the evaporation rate for use in subsequent source-term calculations. This step ensures that the drying kinetics are calculated consistently with the instantaneous cell temperature and humidity. By performing these updates in DEFINE_ADJUST, the model remains fully coupled to the flow solver and responds dynamically to changes in local thermodynamic conditions. The DEFINE_SOURCE macro is used to introduce additional mass and energy source terms into the governing equations. In the context of drying, a mass source term is added to the vapour species transport equation, representing the water mass released during drying. A corresponding energy sink is added to the energy equation, accounting for the latent heat of vaporisation.

Both source terms utilise the evaporation rate calculated earlier in the DEFINE_ADJUST macro, thereby ensuring thermodynamic consistency. Fluent evaluates these source terms at each iteration, allowing the solver to account for local drying effects such as cooling and the generation of water vapour.

A mass source term is added to the continuous phase (air), from the porous zone of the mushrooms to account for the vaporisation of moisture from the mushrooms, which is governed by Fick’s second law of diffusion^53^.

5 $$\:Sm=\:-\:\left(1\:-\:\epsilon\:\right)\text{*}{\rho\:}_{m}\frac{dMt}{dt}$$

Where.

ρ_m_ is the density of the mushrooms, and ε is the porosity of the mushrooms.

The changes in momentum caused by the porosity of the mushrooms were calculated by using the porous media model in Fluent, which requires the porosity, which was specified at 0.38^52^ and the viscous and inertial resistance values. These are obtained as 12,924 $\:{m}^{-1}$ and 1.444e-8 $\:{m}^{-2}\:$using Ergun equation as shown below^51^:

6 $$\Delta P/L = \left[ {150{\text{*}}{{\left( {1 - \:} \right)}^2}{\text{*}}\mu \:{\text{*}}U} \right]/\left( {{\:^3}{\text{*}}d{{\text{?}}^2}} \right) + \left[ {1.75{\text{*}}\left( {1 - \:} \right){\text{*}}\rho \:{\text{?*}}{U^2}} \right]/\left( {{\:^3}{\text{*}}d{\text{?}}} \right)$$

Where,

ΔP = pressure drop, L = Length of food sample, ε = Porosity, µ = Dynamic viscosity, U = Velocity, ρ_m_ = Density of the media, dp = Effective diameter of the media^52^.

The source term in the energy equation is added to account for the energy loss due to evaporative cooling and is determined using

7 $$\:\phi\:\:=\:-{h}_{fg}(1-\epsilon\:){\rho\:}_{m}\frac{dM}{dt}$$

Where, $\:{h}_{fg}$ = latent heat of vaporisation.

The value of $\:\frac{dM}{dt}$ calculated using the mass source term was substituted in the equation of the energy source term.

### Analysis of the thermo-hydraulic characteristics

The thermal and hydraulic performance of the SAH duct is characterised using non-dimensional parameters such as Nu and f.

The hydraulic diameter of the pipe plays a crucial role in determining the inlet Reynolds number.

8 $$\:Re=\frac{\rho\:UD}{\mu\:}$$

The average temperature over the area of the collector plate, along with the average bulk average temperature, is derived from simulation results. The mean heat transfer coefficient and average Nu are calculated using.

9 $$\:h=\frac{q}{({T}_{p}-{T}_{b})}$$

10 $$\:Nu=\frac{hD}{k}$$

The friction factor (f) is calculated to assess the hydraulic efficiency of SAH.

11 $$\:f=\frac{\varDelta\:PD}{2\rho\:L{U}^{2}}$$

Heat transfer can be enhanced by incorporating artificial roughness, which invariably results in an accompanying increase in pressure drop, necessitating greater pumping power. Consequently, to validate the implementation of ribs in solar air heaters (SAH), it is essential to establish a balance between the improvement in heat transfer and the associated pressure drop. In the context of SAHs, the efficacy of ribs is assessed through the thermo-hydraulic performance parameter^54^.

12 $$\:THPP=\frac{{(Nu}_{r}/Nu)}{{{(f}_{r}/f)}^{1/3}}$$

To study the drying kinetics, the page model is used for this study as it predicts the drying characteristics of the specimen (mushroom) with better accuracy^55^.

13 $$\:\:\:\:\:\:\:\:\:\:\:\:\:\:\:\:\:\:\:\:\:\:\:\:\:\:\:\:\:\:\:\:\:\:\:\:\:\:MR=\text{exp}\left(-k{t}^{n}\right)$$

The moisture content present in the sample and its variation with time is given by^52^

14 $$\:MR=\:\frac{{M}_{t}-{M}_{e}}{{M}_{o\:}-\:{M}_{e}}$$

Where,

$\:{M}_{t}$ = Moisture content at time t, $\:{M}_{e}$ = Equilibrium moisture content, $\:{M}_{o\:}$ = Initial moisture content.

The sample drying rate is computed by taking the time derivative of the moisture ratio using

15 $$\:\frac{dMt}{dt}=\frac{dMR}{dt}({M}_{o\:}-\:{M}_{e})$$

Where equilibrium moisture content changes with the current moisture content of the mushroom, therefore, $\:{M}_{e}$ is calculated using moisture sorption models.

The equilibrium moisture content at each time step can be determined by using a moisture sorption model that describes the thermodynamic relationship between the water activity and equilibrium moisture content at a given temperature and pressure. The Modified Henderson model is used to compute equilibrium moisture content^56^.

16 $$\:{M}_{e}=\:-\frac{\text{l}\text{n}{(1-\:{a}_{w})}^{1/C}}{A(T+B)}$$

Where A, B, and C are model constants having values − 0.000952, −43.8 and 1.36, respectively, for the operating temperature considered.

As the drying process in solids depends on the diffusion of water in the solid, the moisture ratio (MR) of the product at an instant during the drying process is expressed in terms of the effective diffusion coefficient (D_e_)^57^

17 $$\:\:\:\:\:MR=\frac{8}{{\pi\:}^{2}}exp\left(\frac{-{\pi\:}^{2}{D}_{e}t}{4{B}^{2}}\right)$$

### Validation of the numerical methodology

**Fig. 5:**
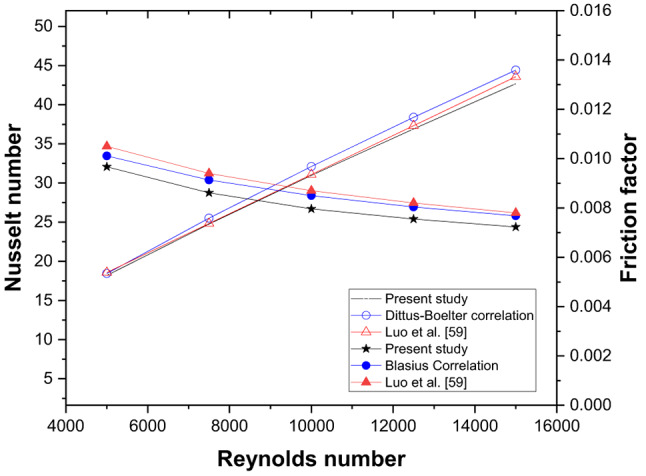
Validation of smooth plate triangular duct solar air heater.

**Fig. 6:**
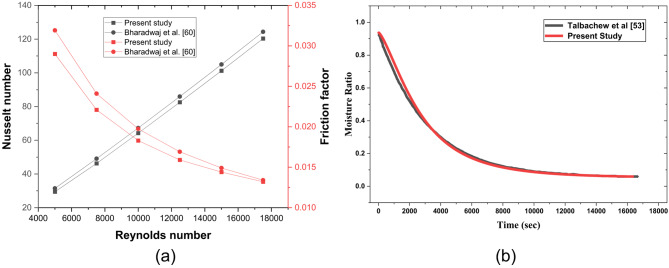
(**a**). Comparison of ribbed triangular duct CFD model with experimental results^59^, and (**b**). Validation of Dryer numerical model.

To assess the validity of the computational fluid dynamics (CFD) methodology, a comparison was conducted with an investigational study^58^ and theoretical correlations, namely the Dittus-Boelter equation and the altered Blasius correlations. The CFD results demonstrated average discrepancies of approximately 2.9% for the Nusselt number (Nu) and 5.21% for the friction factor (f) when compared to the theoretical correlations (Fig. 5). Upon assessment of the experimental study, deviations of 2% for Nu and 7.1% were noted. Therefore, the CFD analysis employed is appropriate for examining the thermo-hydraulic flow characteristics within the triangular duct. For ribbed TSAH, the numerical simulation results are validated with for e = 4 mm and P/e = 5^59^ as shown in Fig. 6a, resulting in a maximum deviation of 7% and 10%, respctively, for *Nu* and *f.* The numerical methodology adopted to simulate the drying mechanism in the indirect-type solar dryer is validated, yielding a maximum deviation of 6% (Fig. 6b) in the variation of moisture ratio with time during the initial drying period^60^.

## Results and discussion

This 3-D numerical study elucidates the synergistic effect of a pin fin with a discrete V-rib on the thermal and hydraulic characteristics of a triangular duct solar air heater (TSAH). The effects of P/e (5 to 15) and g/e (0.2 to 0.8) are studied, along with solid and porous pin-fins of 2 mm diameter (equal to the optimal gap width), on the thermal and hydraulic characteristics of TSAH in the turbulent flow regime (5000 ≤ Re ≤ 15000). The effect of secondary flow generated by V-rib and its synergistic effect in the presence of a pin-fin are expressed in the form of characteristic plots, contours, and streamlines for better clarity. With the design modifications inducing additional turbulence, paving the way for better performance and higher outlet air temperatures, is further analysed from the application perspective of an integral indirect type dryer suitable for medium temperature drying applications. The drying rate of mushrooms is analysed and compared with all four SAH configurations using variations in moisture ratio, drying rate, and diffusion coefficient over time.

### Nusselt number and friction factor characteristics of continuous ribs

**Fig. 7:**
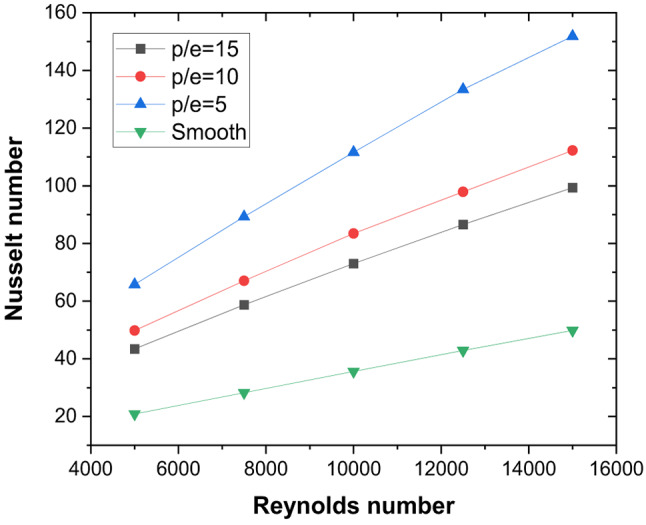
Effect of Reynolds number on heat transfer characteristics of continuous V-ribs of varying pitch.

The effect of flow rate (or Reynolds number) is studied for a constant rib height (e) and inclination of 5 mm and 45°, respectively, with pitch (P) varying in the range of 5 < P/e < 15. With an increase in Reynolds number (Re), a monotonous increase in heat transfer is observed for continuous V-ribs (Fig. 7) with a higher Nusselt number (Nu) compared to a smooth plate absorber in a triangular duct solar air heater (TSAH). With increased flow velocity, boundary layer thickness decreases, resulting in a thinner laminar sublayer. As a result, the resistance to heat transfer from the hot absorber plate to air decreases, leading to higher heat transfer compared to lower flow velocity in smooth plate TSAH. The velocity gradients near the absorber plate become steeper as the Reynolds number (Re) increases. This leads to more significant velocity fluctuations and amplifies the turbulence in the flow owing to larger inertial forces. As the inertial force dominance substantially increases over the viscous force, more inertial energy is available for conversion into turbulence, resulting in higher turbulent kinetic energy (TKE).

**Fig. 8:**
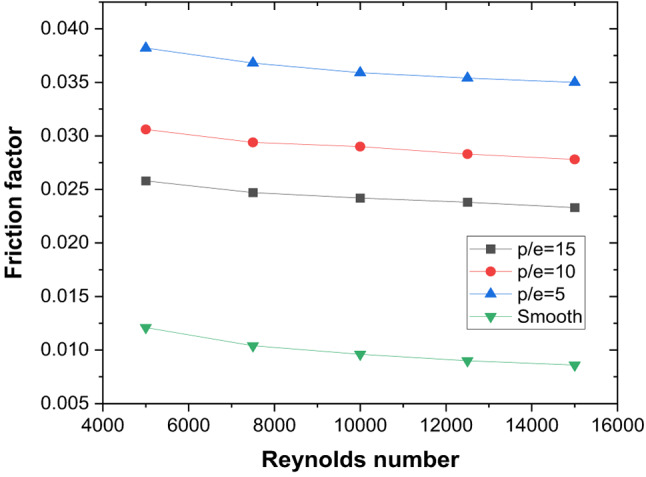
Effect of Reynolds number on friction factor characteristics of continuous V-ribs of varying pitch.

With artificial roughness (V-ribs) underneath the absorber plate, flow separation happens; as a result, the number of localised reattachment points on the absorber plate increases. The average Nu increases with the increase in localised reattachment points on the absorber plate, resulting in improved heat transfer compared to the smooth plate. In addition, the secondary flow created on the limbs of the V-ribs interacts to cascade energy to smaller scales, increasing the TKE. As the flow velocity increases, stronger eddies are formed in the region between the ribs owing to the coalescence of secondary flow at the apex of the V-rib. It is observed that maximum Nu is obtained for P/e = 5, and as P/e increases, the enhancement in Nu decreases compared to the smooth duct (Fig. 7). Whereas, as pitch increases, the number of V-ribs underneath the absorber plate decreases, resulting in lower flow obstruction at higher P/e. This results in more recirculation zones in the wake region of the ribs. Hence, as P/e increases, the incremental pressure drop compared to the smooth duct decreases (Fig. 8). However, the rate of decrease reduces from P/e = 10 to P/E = 15.

**Fig. 9:**
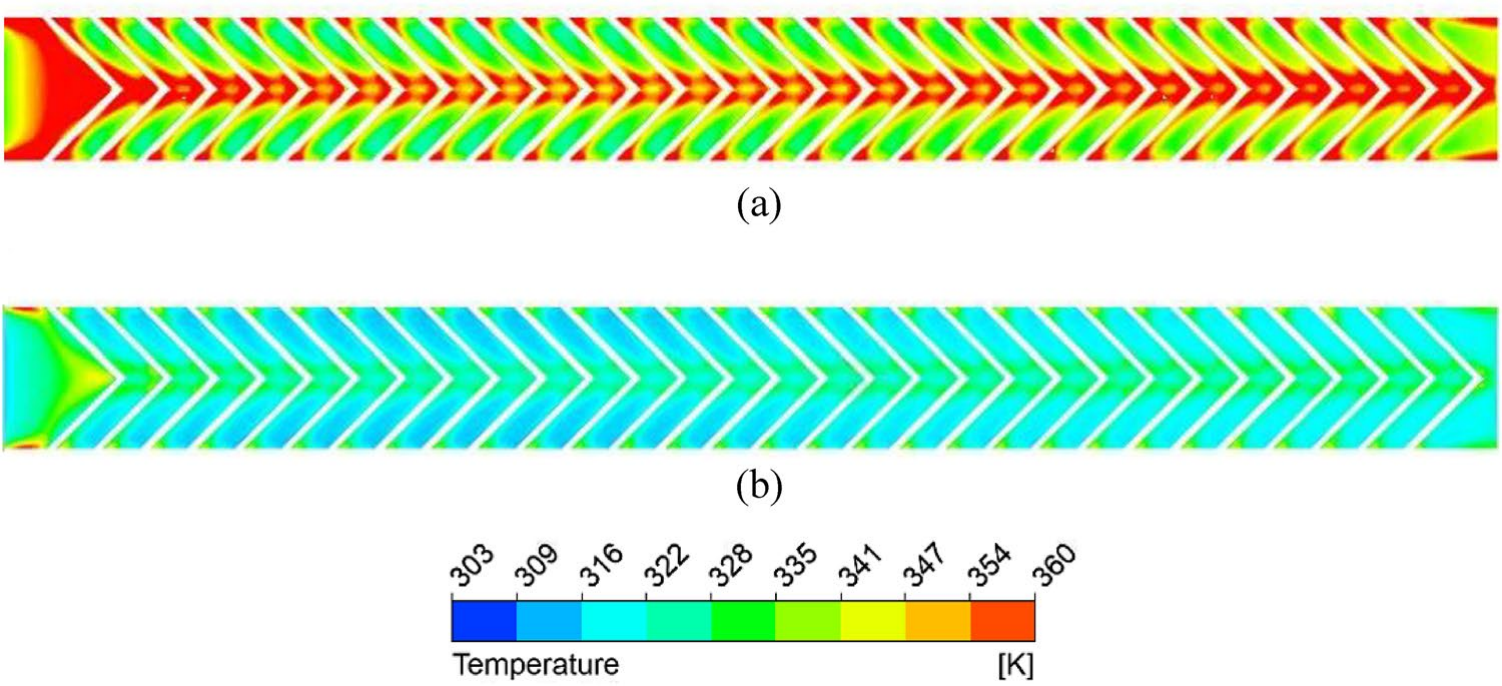
Absorber plate temperature distribution for (**a**) Re = 5000 and (**b**) Re = 15,000.

**Fig. 10:**
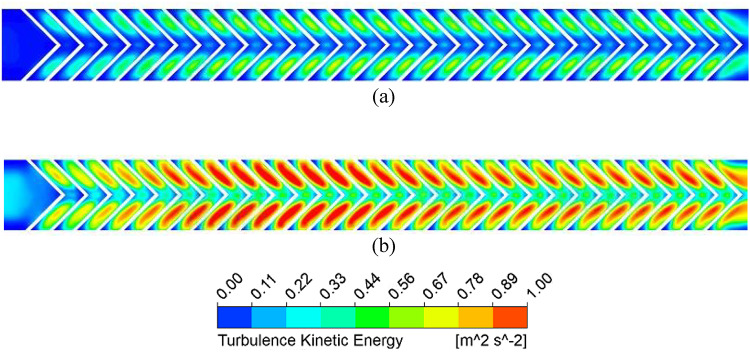
Turbulence kinetic energy distribution for (**a**) Re = 5000 and (**b**) Re = 15,000.

Figure 9 shows that the absorber plate temperature decreases at higher Reynolds numbers (Re) due to a thinner thermal boundary layer, indicating a higher convective heat transfer coefficient. High-temperature zones persist near the sidewalls and at the V-rib apex, where secondary flows from both limbs converge. Figure 10 further confirms that the secondary flow carries higher energy along the rib surfaces due to the helical motion of fluid from the sidewalls toward the centre. At the apex, turbulent kinetic energy (TKE) reduces slightly due to mixing of hotter and colder fluid layers.

**Fig. 11:**
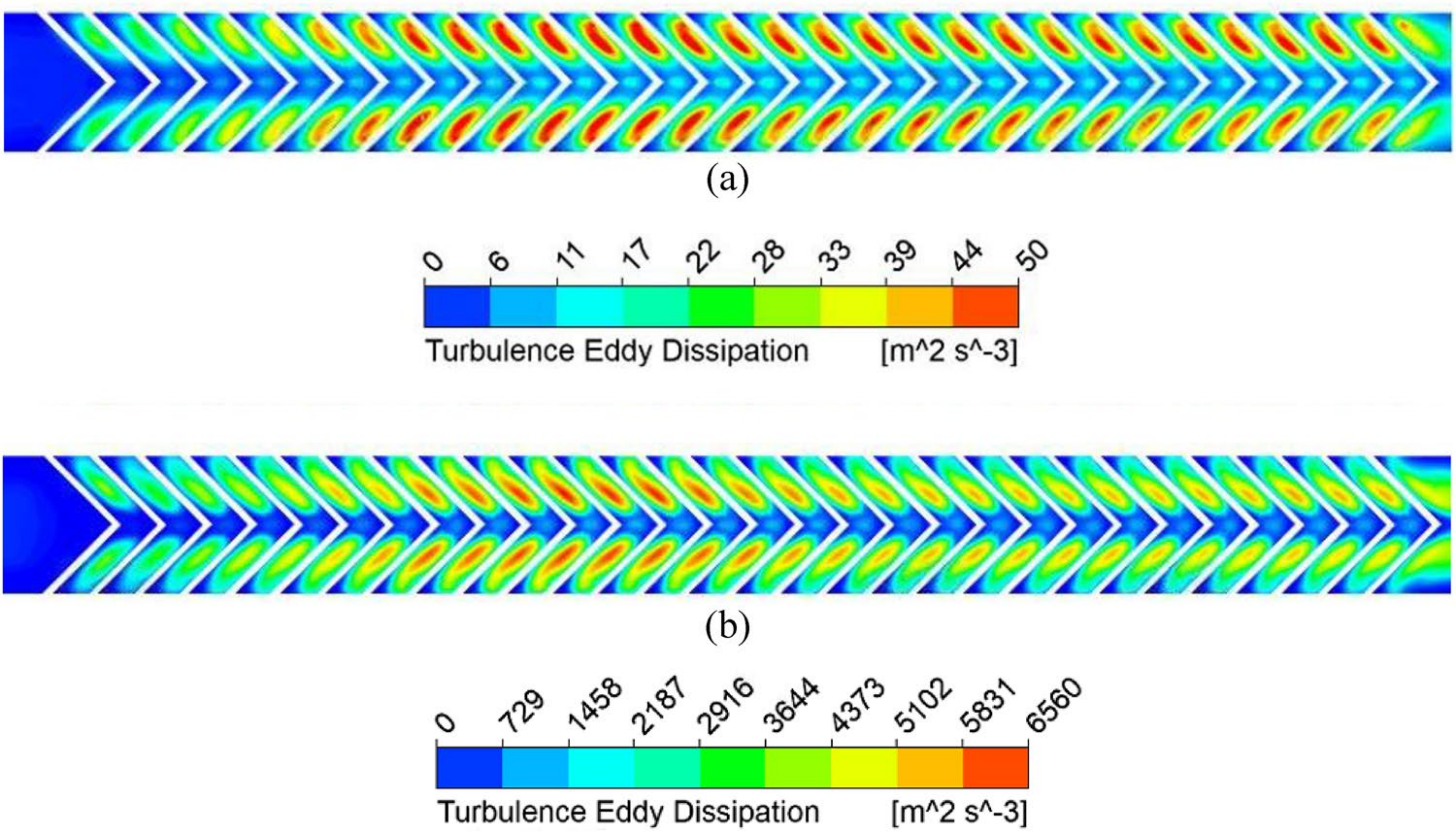
Turbulence eddy dissipation along the absorber plate length for (**a**) Re = 5000 and (**b**) Re = 15,000.

**Fig. 12:**
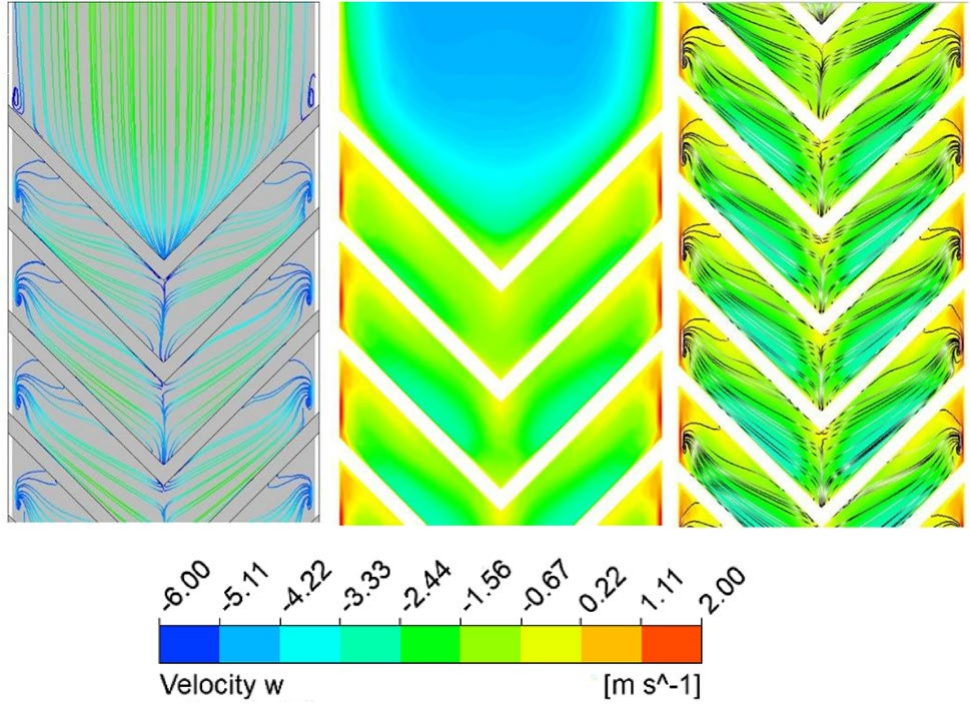
Streamlines and velocity component along the ‘z’ direction (secondary flow) flow intensity for Re = 15,000.

TKE dissipates into thermal energy through the turbulent eddy dissipation rate (TED), which represents the transfer of energy from larger eddies to smaller ones. Higher TED enhances energy cascading, disrupts the thermal boundary layer, and increases the convective heat transfer coefficient. However, stronger eddy dissipation also increases pressure drop due to loss of kinetic energy. Figure 11 shows a higher TED at larger Re, especially along the rib surfaces, where transverse motion dominates. From streamline patterns (Fig. 12), secondary flow velocity is highest at the rib leading edge and decreases toward the apex; it rises again after merging and being carried to the next rib.

**Fig. 13:**
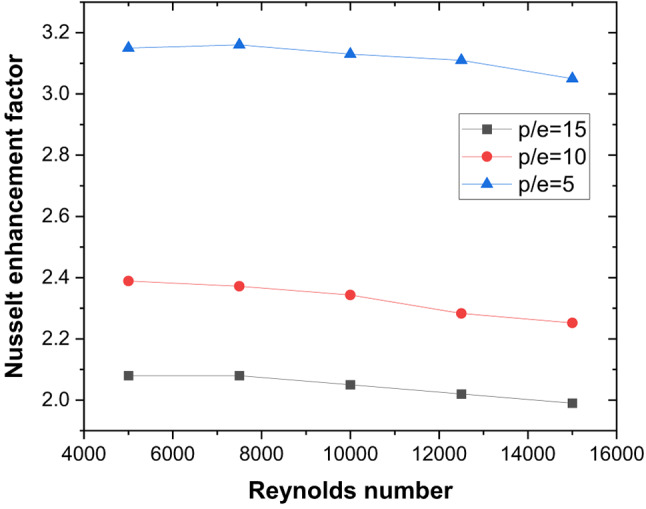
Nusselt number enhancement for various rib pitches.

**Fig. 14:**
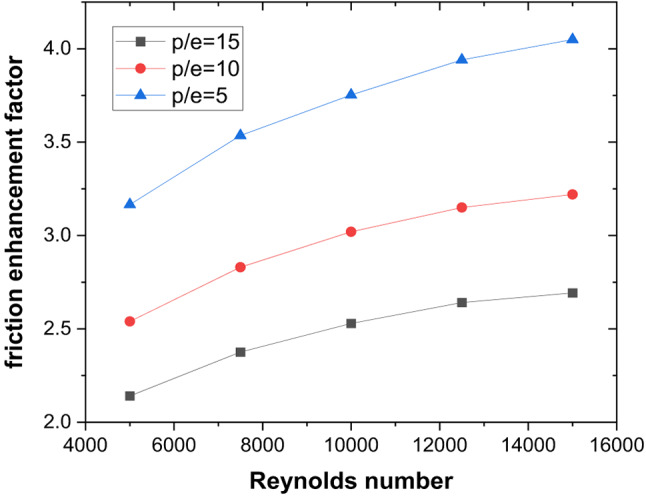
Friction factor enhancement for various rib pitches.

**Fig. 15:**
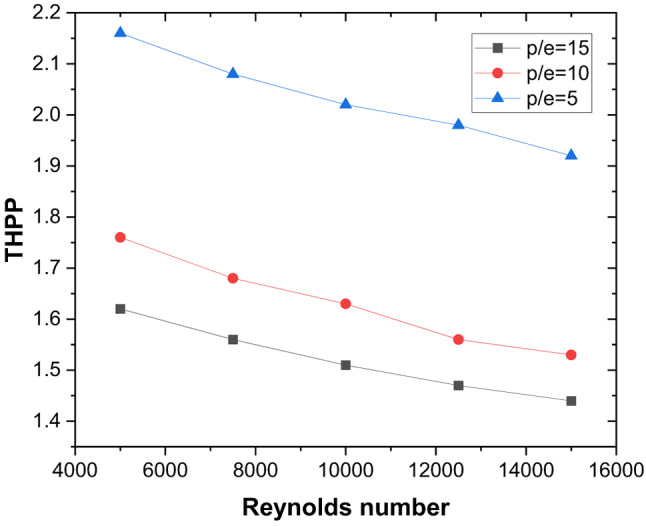
Thermo-hydraulic performance parameter variation for different rib pitches and Reynolds number.

These thermal–hydraulic mechanisms yield a maximum Nusselt number enhancement of 3.15 times that of the smooth duct at P/e = 5, Re = 5000 (Fig. 13). The friction factor increases by 3.17 times (Fig. 14), resulting in a maximum THPP of 2.16. THPP decreases with increasing P/e and Re (Fig. 15).

### Nusselt number and friction factor characteristics of discrete V-ribs

**Fig. 16:**
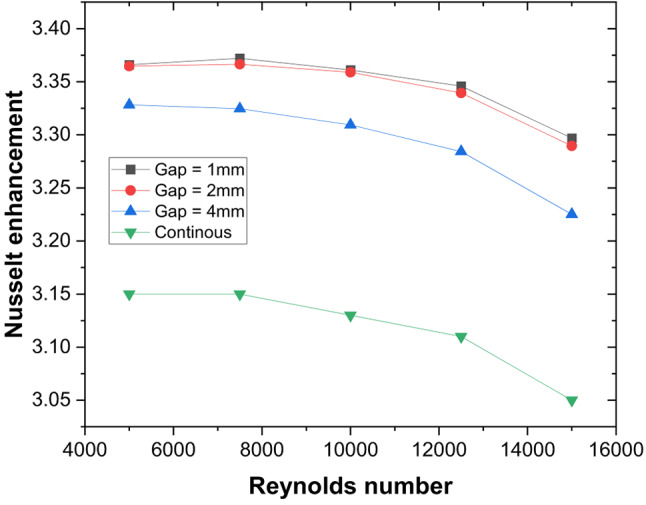
Nusselt number enhancement for various gap widths in discrete V-rib.

**Fig. 17:**
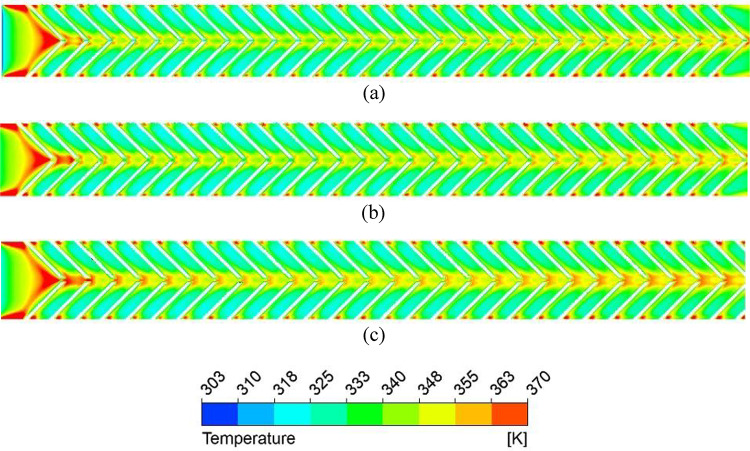
Absorber plate temperature distribution for various gap widths (**a**) g = 1 mm, (**b**) g = 2 mm, and (**c**) g = 4 mm.

**Fig. 18:**
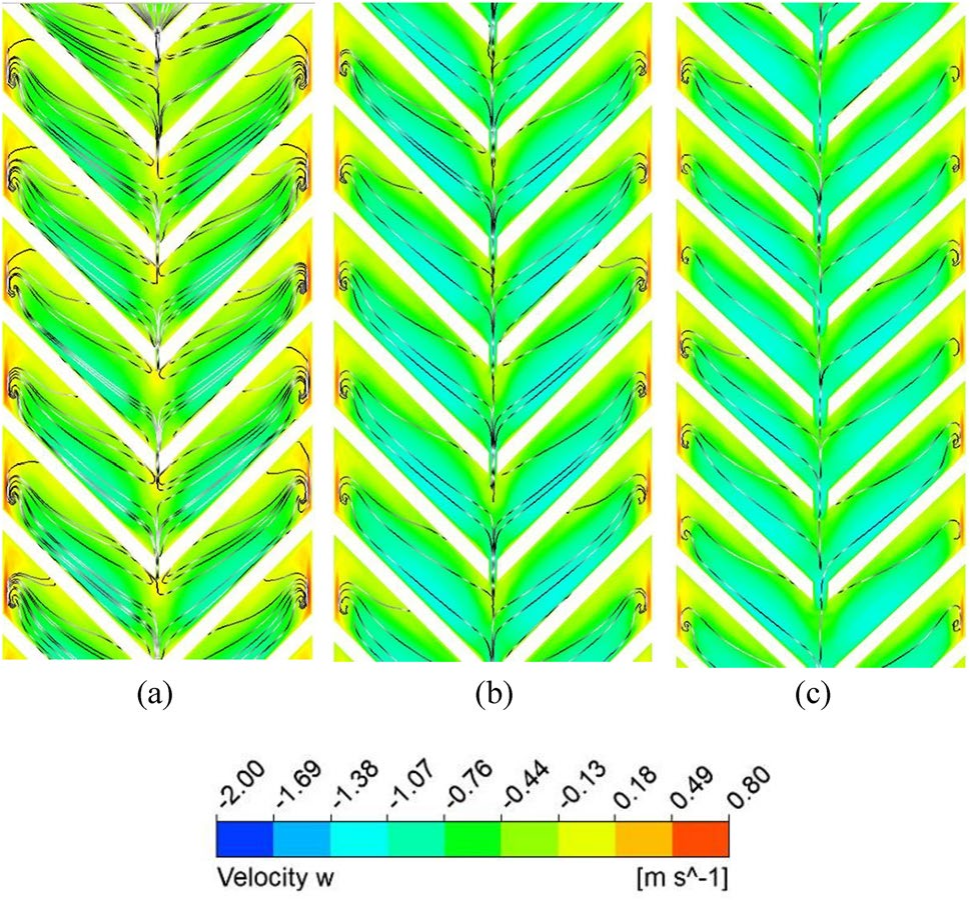
Secondary flow pattern for (**a**) g = 1 mm, (**b**) g = 2 mm, and (**c**) g = 4 mm.

**Fig. 19:**
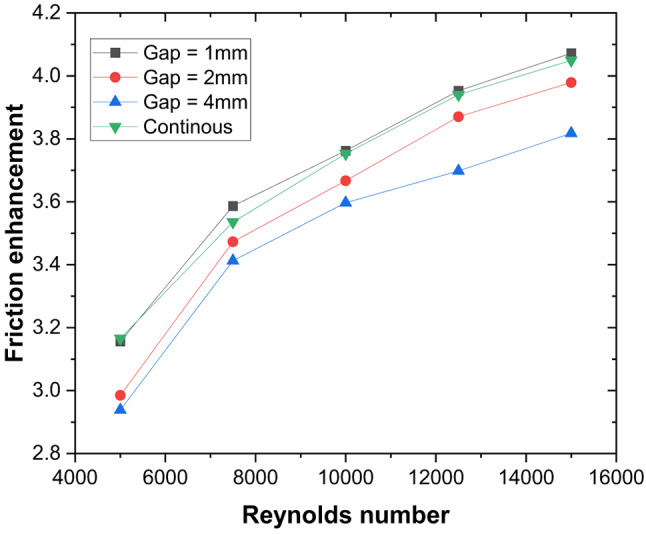
Friction factor enhancement for various gap widths in discrete V-rib.

**Fig. 20:**
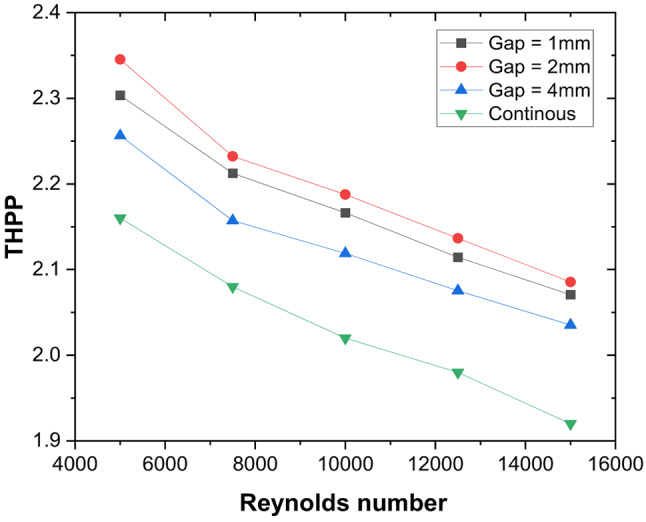
Thermo-hydraulic performance parameter variation for various gap widths and Reynolds numbers.

A gap is introduced to eliminate the higher temperature zones at the apex of the ribs (P/e = 5) created by the coalescence of two secondary flows, and the effect of width (g) on the thermo-hydraulic performance is studied. Due to the gap at the apex of the V-rib, enhancement in Nu increases to 3.36 for P/e = 5 at Re = 5000 (Fig. 16). The enhancement in Nu is attributed to the following reasons. Gaps in the V-ribs allow the fluid to separate and reattach downstream of the gap. Flow reattachment creates localised turbulence near the rib apex, significantly enhancing the heat transfer rate within the reattachment zone. Further, gaps introduce localised vortices and additional secondary flow structures at the edges of the gaps. These vortices enhance mixing between the core flow and the near-wall region, thereby reducing the absorber plate temperature (Fig. 17) and promoting improved convective heat transfer. In addition, owing to flow passage through gaps (Fig. 18), the flow obstruction is less than continuous V-ribs, resulting in a lower friction factor (Fig. 19). Maximum THPP of 2.30 is obtained for a gap width of g/e = 0.4 at Re = 5000. With an increase in g/e beyond 0.4, THPP does not increase significantly (Fig. 20). This is due to a reduction in the Nu number as the velocity of the mainstream flow through the gap decreases, resulting in less energy transfer to the secondary flow compared to g/e = 0.4. With an increase in Re, THPP decreases for the range of g/e studied due to higher pressure drop.

### Thermo-hydraulic performance comparison of solid and porous pin fin with discrete V-rib

**Fig. 21:**
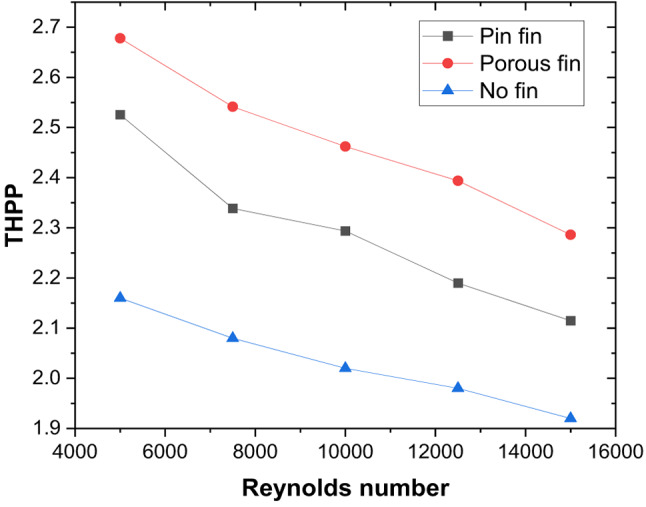
Thermo-hydraulic performance parameter enhancement in discrete V-rib with porous and solid pin fin compared to no-pin fin case.

**Fig. 22:**
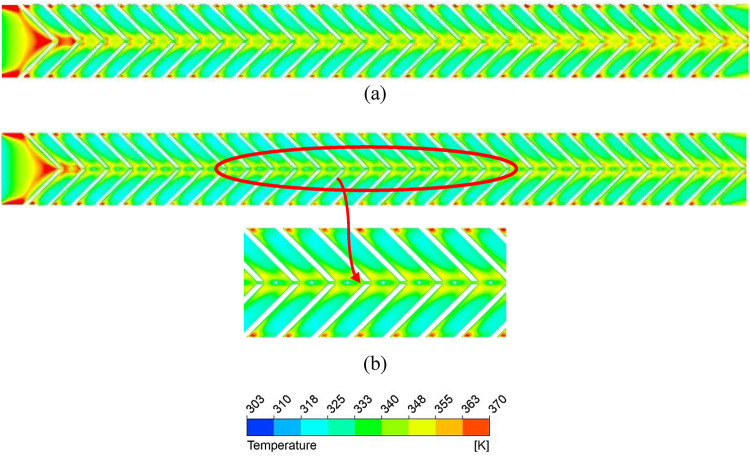
Absorber plate temperature distribution in discrete V-rib (g/e = 0.4) with (**a**) no pin-fin and **(b**) pin-fin.

**Fig. 23:**
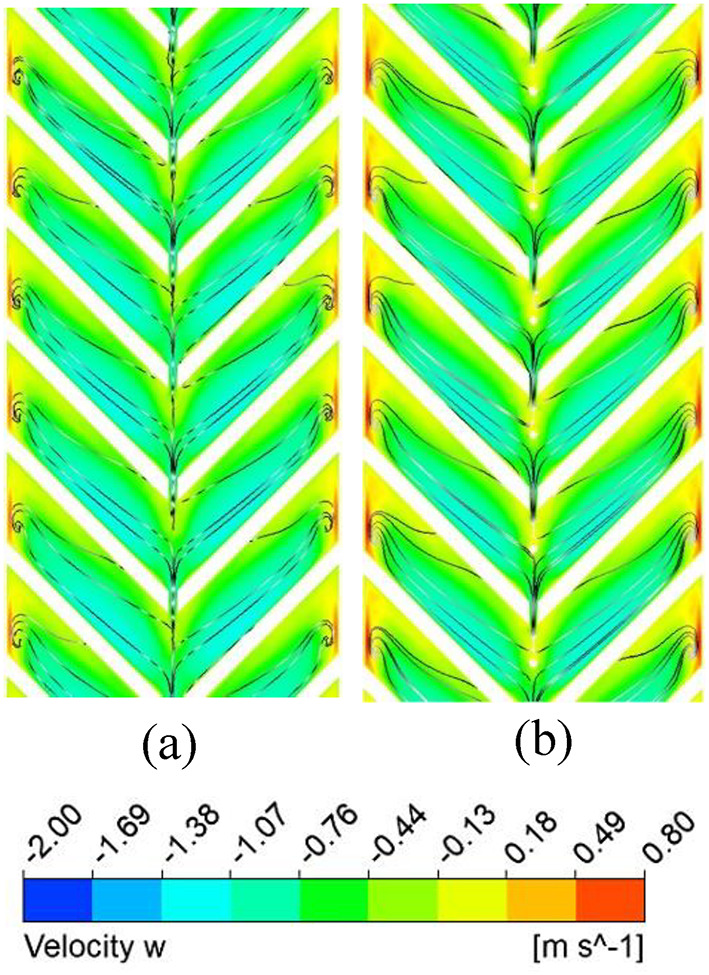
Secondary flow pattern for (**a**) no pin-fin and (**b**) with pin-fin.

With the presence of a gap, as heat transfer improves, the effect of a pin-fin with a 2 mm diameter and height in the inter-rib region (solid and porous) is studied to take advantage of the synergistic effect of vortex interaction with pin fins, which could potentially create additional turbulence and mixing. It is noted that the THPP of the TSAH increases with the presence of pin-fins (Fig. 21). V-ribs introduce the secondary flow, while pin fins increase the surface area and fluid mixing. A maximum THPP of 2.7 is obtained for the discrete V-rib and porous pin-fin at Re = 5000, compared to 2.5 and 2.2 for the solid and no pin-fin cases, respectively. The enhancement in heat transfer is attributed to lower plate temperature near the apex of the V-ribs than the no-fin case (Fig. 22) due to the reduction of mixing region size immediately after the apex (Fig. 23). With porous pin-fins, a larger surface area and numerous internal pores allow fluid passage, increasing thermal interaction with the pin-fin and reducing the thermal gradient with higher bulk temperature. Additionally, the wake region downstream of the pin fin is absent in porous fins, thereby reducing the additional pressure drop.

### Comparison of THPP performance of various previous studies

In this section, the overall performance of various similar ribs in TSAH is compared to the existing literature to elucidate the superior performance of the discrete V-rib with pin-fins under similar operating conditions (Table 6).

**Table 6:** Comparative thermo-hydraulic performance parameter.

Aauthors	Aartificial roughness	Ooptimal geometrical parameter	Rreynolds number	Thpp
Nidhul et al^47^.	Continuous V-rib	P/e = 10, e/D = 0.05	5000 to 20,000	2.1
Misra et al^20^.	V ribs with multiple gaps	P/e = 10, e/D = 0.04	4000 to 20,000	2
Agarwal et al^60^.	Discrete V-rib	P/e = 12	4000 to 20,000	2.2
Present study	Discrete V-rib with pin-fins	P/e = 5; g/e = 0.4, d = 2 mm	5000 to 15,000	2.7

### Indirect-type solar drying application

Drying is a critical post-harvest process that significantly affects the shelf life, quality, and safety of agricultural products. The efficiency and effectiveness of the drying process are strongly influenced by the thermal and flow characteristics of the air supplied to the dryer, which, in turn, are governed by the SAH design. Therefore, integrating the SAH with an indirect-type solar dryer and analysing key drying parameters such as moisture ratio versus time, drying rate versus moisture ratio, and effective moisture diffusion coefficient versus time provides a comprehensive assessment of the system’s practical performance. This approach not only validates the theoretical and numerical findings but also demonstrates the direct impact of SAH design on the quality and kinetics of the drying process for mushroom samples.

**Fig. 24:**
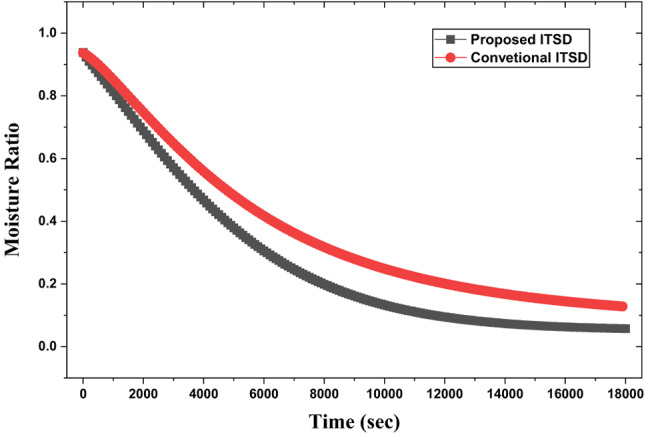
Moisture ratio variation with drying time.

**Fig. 25:**
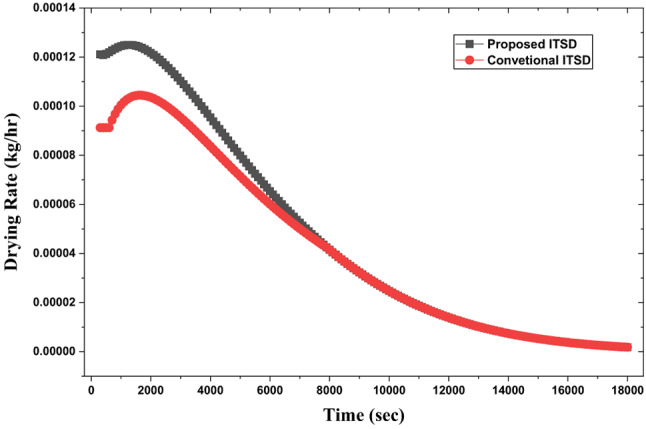
Drying rate variation with drying time.

As triangular duct SAH with pin-fins and V-ribs delivers air at a higher outlet temperature owing to higher intensity secondary flow and turbulence relative to smooth plate SAH in conventional ITSD, the evaporation of surface moisture from the food samples is accelerated, especially during the falling rate period of drying, which is typically controlled by internal diffusion (Fig. 24). This is attributed to high initial water content, which is on the surface of the food samples and the porous structure facilitates rapid initial evaporation. As the mushroom dries further, the remaining water is inside the cells and requires diffusion, a slower process, causing the curve to flatten as it approaches the equilibrium moisture content. Compared to ITSD with conventional smooth SAH, a 66% reduction in drying time is observed with the new SAH design. Upon analysing the drying rate, it is observed that with higher air temperature, the initial drying rate is higher (Fig. 25) and subsequently drops. As discussed earlier, higher air temperatures increase the evaporation of water from the surface during the initial drying period, while lower air temperatures have the same effect at a relatively slower pace. After the surface evaporation, internal diffusion of water occurs at a lower speed as the moisture content approaches the equilibrium level retarding the drying rate.

**Fig. 26:**
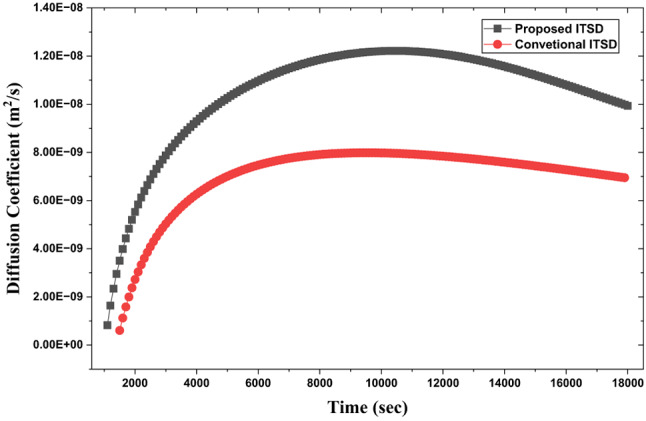
Diffusion coefficient variation with drying time.

The variation of the effective diffusion coefficient during mushroom drying with the proposed and conventional ITSD design can be attributed to the interplay of temperature, moisture content, and structural changes in the material. During drying, water migrates from the interior to the surface mainly by passive diffusion along a concentration gradient. As temperature increases, water molecules acquire greater kinetic energy, resulting in a higher mass transfer rate; at elevated temperatures, these molecules are also less tightly bound to the food matrix, making them easier to remove. This explains why the diffusion coefficient is consistently higher for the proposed ITSD design compared to the conventional (Fig. 26). At the onset of drying, effective diffusivity rises rapidly due to the abundance of free water and the open, porous structure of fresh mushrooms, which facilitates internal moisture migration. In isothermal drying, this coefficient often becomes nearly constant during the constant rate period, when surface evaporation dominates and water vapor diffuses readily from the surface. As drying progresses into the falling rate period, the internal diffusion of liquid water becomes the predominant mechanism, and the diffusion coefficient starts to decline.

## Conclusion

TSAH with discrete V-ribs has been studied along with pin-fins in the inter-rib region to analyse the synergistic effect of secondary flow and pin-fins on thermo-hydraulic performance. This design exhibits improved thermohydraulic performance compared to similar designs. With the hot air supplied by the ribbed triangular duct solar air heater (SAH), the drying characteristics of mushrooms are studied and compared to the conventional ITSD. This not only validates the numerical findings but also demonstrates the direct impact of SAH design on the quality and kinetics of the drying process for mushroom samples. The significant observations of the analysis are as follows:

- Discrete V-ribs enhance the heat transfer rate over the continuous V-ribs owing to additional turbulence and mainstream flow energising the secondary flow merging at the apex from either limb of the ribs.
- Maximum enhancement in Nu and f is obtained as 3.36 and 2.98, respectively, resulting in a THPP of 2.35 for g/e = 0.4 at Re = 5000.
- With the synergistic effect of pin-fin and discrete V-ribs, a maximum THPP of 2.68 is obtained with porous pin-fins of similar height at Re = 5000 compared to 2.25 obtained with no-fin.
- The proposed ITSD (with ribbed triangular duct SAH) outperforms the conventional ITSD, demonstrating improved drying performance. Compared to ITSD with conventional smooth SAH, a 66% reduction in drying time is observed due to the higher air outlet temperature. Additionally, the effective diffusivity coefficient of food samples increased by 54% relative to that of conventional ITSD.

These results can aid in the development of advanced drying technologies, improve modelling accuracy, and scale up industrial applications. Furthermore, the outcomes of this work align with the United Nations Sustainable Development Goals (SDGs), particularly those related to clean energy, innovation in food processing, and reducing food waste through improved drying methods.

## Data Availability

The data that support the findings can be provided upon request to the corresponding author.

## Abbreviations

CFD: Computational fluid dynamics
C_p_: Specific heat capacity, J/kg. K
D: Hydraulic diameter of the duct, mm
D_e_: Equilibrium diffusion coefficient, m^2^/s
e: Rib height, mm
EWT: Enhanced wall treatment
f: Average friction factor
h: Average heat transfer coefficient, W/(m^2^.K)
H: Duct height, mm
ITSD: Indirect-type solar dryer
k: Thermal conductivity of air, W/(m.K)
$\:{k}_{e}\:$: Effective thermal conductivity
L: Test section length, mm
M: Moisture content
MR: Moisture ratio
Nu: Average Nusselt number
P: Rib pitch, mm
q: Heat flux, W/m^2^
Re: Reynolds number
R_h_: Relative baffle height
RNG: Renormalized group
THPP: Thermo-hydraulic performance parameter
*R*_p_: Relative baffle pitch
SAH: Solar air heater
*T*_b_: Bulk mean temperature of air, K
TKE: Turbulence kinetic energy
LTE: Local thermal equilibrium
GCI: Grid convergence index
TED: Turbulent eddy dissipation
*U*: *U*Inlet velocity of air, m/s
*W*: Duct width, mm
Δp: Pressure drop, Pa
m: Flow rate of air kg/m
*T*_o_: Outlet temperature of the air, K
T_i_.: Inlet temperature of the air, K
*T*_p_: Average temperature of the absorber, K

## Greek symbols

μ: Dynamic viscosity of air, Pa.s
ρ: Density of air, kg/m3
φ: Porosity

## Subscript

b: Bulk
e: Equilibrium
h: Height
P: Pitch
p: Plate
r: Roughened
